# Evaluation of the effectiveness of psychosocial rehabilitation: an innovative approach based on the analytic hierarchy process

**DOI:** 10.1192/j.eurpsy.2024.409

**Published:** 2024-08-27

**Authors:** V. Mitikhin, T. Solokhina

**Affiliations:** ^1^Mental Health Research Centre, Moscow, Russian Federation

## Abstract

**Introduction:**

In psychosocial rehabilitation (PSR), rank scales are widely used to assess the severity of functional disorders in patients. The main problem of evaluating the effectiveness of PSR is related to the methods of processing data obtained using psychometric scales used to evaluate the effectiveness of interventions within PSR. The proof of the non-numerical nature of ranks was obtained by J. Pfanzagl (1968). Incorrect processing of rank information obtained in the framework of psychosocial research leads to contradictions in the assessment of the patient’s condition. Consequently, rank processing does not allow classical mathematical operations (summation, average), which makes it impossible to correctly estimate the effectiveness of PSR numerically.

**Objectives:**

Development of algorithms for numerical evaluation of PSR efficiency based on rank information processing using the analytic hierarchy process (AHP) [1].

**Methods:**

Clinical, psychometric, AHP algorithms

**Results:**

The analysis of the problems of assessing the patient’s conditions on the basis of categorical and psychometric (rank) scales and subscales shows that these problems can be presented in the form of appropriate hierarchies, the structure of which must be taken into account when processing the initial information.

According to the results of the analysis of the data of preliminary studies, the main areas of impaired functioning of patients affecting the evaluation of the effectiveness of PSR have been identified. Rank estimates of changes in the relevant areas of the patient’s dysfunction after the PSR program compared to the initial level are the basis for the conclusion about the effectiveness of the PSR components. Algorithms of the AHP normative approach were used to translate rank information into numerical information [2]. The weight of the areas of the patient’s functioning disorders was used in the formation of integral estimates of the effectiveness of PSR.

The fundamental difference between AHP-based assessments and rank assessments is due to the fact that numerical estimates of the weight of the criteria and the corresponding changes in the patient’s condition are obtained, which depend on the qualifications of specialists, the characteristics of the scales used to measure violations in the relevant areas and the procedures of the PSR.

**Conclusions:**

Obtaining the results of processing rank information in a numerical scale allows to obtain the correct integration of the patient’s personal characteristics when considering PSR procedures and to obtain correct prognostic models of the patient’s condition. 1. Saaty T. European Journal of Operational Research.1990; 48(1):9-26. https://doi.org/10.1016/0377-2217(90)90057-I 2. Mitikhin V.G., Solokhina T.A. et al. Psychiatry, 2022; 20(2): 51-59. DOI: 10.30629/2618-6667-2022-20-2-51-59

**Disclosure of Interest:**

None Declared

